# Frailty Status Among the Elderly of Different Genders and the Death Risk: A Follow-Up Study

**DOI:** 10.3389/fmed.2021.715659

**Published:** 2021-08-13

**Authors:** Jing Shi, Yongkang Tao, Li Meng, Baiyu Zhou, Chunbo Duan, Huan Xi, Pulin Yu

**Affiliations:** ^1^Beijing Institute of Geriatrics, Beijing Hospital, National Center of Gerontology, National Health Commission, Chinese Academy of Medical Sciences, Beijing, China; ^2^Department of Gastroenterology, China-Japan Friendship Hospital, Beijing, China

**Keywords:** frailty, elderly, different genders, death risk, follow-up study

## Abstract

**Background:** Frailty in the elderly population is currently a frontier and focus in the field of health and aging. The goal of this study was to explore the frailty status among the elderly of different genders and its influence on the risk of death during 11 years.

**Methods:** Frailty index (FI) was used to evaluate the frailty status in the elderly based on the baseline data conducted in 2009; and death as outcome variables collected in 2020 were analyzed. The difference of the frailty level and mortality of different genders was compared. Cox regression and Kaplan–Meier curves were applied to evaluate the influence on the risk of death and the 11-year survival of the elderly at different level of frailty, respectively.

**Results:** Totally, 1,246 elderly people were recruited. The mortality in men (43.7%, 227/519) was statistically higher than that in women (34.3%, 249/727) (*x*^2^ = 11.546, *P* = 0.001). Deficits accumulated exponentially with age, and at all ages, women accumulated more deficits than do men on average (*B* = 0.030 vs. 0.028, *t* = 4.137, *P* = 0.023). For any given level of frailty, the mortality rate is higher in men than in women, and the difference in mortality between genders reached the peak when FI value was 0.26. Cox regression analysis showed that FI value had a greater impact on the risk of death in older men (*HR* = 1.171, 95%*CI*: 1.139~1.249)than that in older women (*HR* = 1.119, 95%*CI*: 1.039~1.137). Survival analysis showed that the median 11-year survival time in women was longer than that in men (95.26 vs. 89.52 months, *Log rank* = 9.249, *P* = 0.002). Kaplan–Meier curves showed that the survival rate decreased with the increase of frailty, and at the same level of frailty, survival time in older women was longer than that in older men, except for severe frailty (FI ≥ 0.5).

**Conclusion:** The frailty status and its influence on mortality are different among the older people of different genders; therefore, specific interventions for frailty should be conducted in the elderly population of different genders, as well as of different degrees of frailty.

## Introduction

The study on frailty in the elderly population is currently a frontier and focus in the field of health and aging. The concept of frailty was formally introduced at a meeting of the American Geriatrics Society in 1978 and used to describe a health status in the elderly population with various accumulated health problems requiring long-term support in activities of daily living (1). Since the phenotypic definition of frailty was first introduced by Fried et al. (2) in 2001, the concept of frailty has attracted attention in the field of geriatric medicine, and study publications on frailty have also rapidly increased. Studies have shown that frailty is closely associated with falls, dementia, fractures, and cardiovascular diseases in the elderly population, and the degree of frailty greatly impacts survival rates (3, 4). The degree of frailty in the elderly population has important predictive value for the risks of many adverse outcomes, such as disability, reduced activities of daily living, lengthening of hospitalization, and death events (5, 6).

The frailty index (FI) model, established by Canadian geriatric specialist Professor Kenneth Rockwood and his team, is based on the accumulation of health deficits and currently one of the most popular model to assess frailty in the elderly population (7). The assessment with the FI model successfully achieves a quantitative description of the frailty status in the elderly population and provides an effective way to quantitatively assess the health status of the elderly population. The assessment with FI provides a broader range than other indicators. Multiple health variables can be represented as a single indicator able to reflect a variety of biological processes and more accurately measure the overall health status of the elderly population, enhancing the statistical power of outcome estimation (8). One study demonstrated that the FI outperformed other frailty assessment tools in predicting adverse health prognoses, including death events (9). Due to physiological differences, the progression and manifestation of frailty vary in the elderly population of different genders (10–12). The results of foreign studies based on community population showed that frailty degree in women was higher than in men at the same age, and the mortality rate was higher in men than in women at the same degree of frailty (13). So far, there have been few studies on the FI model in the elderly population of different genders in Chinese communities or on the analysis of the impact of frailty on long-term mortality risk. The elderly population in Beijing urban communities were included in this study, and the prospective analysis utilized the FI model to assess differences in frailty in the elderly population of different genders and the impact on mortality risk, so as to provide a basis for further specific intervention measures to decrease adverse health prognosis caused by frailty.

## Materials and Methods

### Study Setting and Participants

This is a secondary analysis of the Health Status and Fall Status Follow-up Survey database, a representative cohort of community dwelling elder people aged 60 years and older. In this study, the baseline survey population in 2009 were used as samples, and death events from this cohort collected in the follow-up survey in 2020 were used as the outcome variables. The baseline survey was conducted in 2009 in Longtan Sub-district Office jurisdiction of Beijing's Dongcheng District as the survey site using cluster random sampling. A total of 1,578 elderly residents were eligible. The inclusion criteria: the elderly who were ≥60 years during the survey and having lived in the survey site for more than 1 year. The exclusion criteria: the elderly who were not living in the survey site during the survey or were unable to cooperate to complete the survey. A total of 37 elderly residents refused the survey and 63 elderly residents were lost to follow-up (not at home on two visits during the survey). The actual number of survey subjects was 1,536, with a response rate of 95.2%; and a total of 1,512 valid questionnaires were obtained, with a total valid rate of 98.4%. In 2020, a follow-up study on the 1,478 baseline survey population was conducted, and information related to death events among this population was also collected. By 2020, 232 subjects were lost to follow-up, with a lost-to-follow-up rate of 14.0% (232/1,478). All were subjects who left or moved out of the survey site, with an average age of 68.24 ± 3.58 years, including 108 men (46.6%) and 124 women (53.4%). A total of 1,246 subjects were finally included in the analysis, including 519 men (41.7%) and 727 women (58.3%) with an average age of 72.05 ± 4.52 years. The age of the elderly who were lost-to-follow-up was lower than those included in the study (*t* = 12.148, *P* = 0.000), but there was no significant difference between both genders (χ^2^ = 1.921, *P* = 0.166). All subjects signed the informed consent forms.

### Study Content and FI Construction

In this study, a standard questionnaire validated by multiple rounds of expert discussion was used and information was collected by face-to-face interview. The information included demographic characteristics, social support and economic status, health and physical status, diseases and signs, medications, cognition and emotion stats, balance function test, activities of daily living assessment (ADL, IADL), and comprehensive geriatric assessment. Based on the content of the questionnaire, a total of 37 variables were selected as the health deficits for calculating the FI according to the criterion of health deficits and standard procedure to construct the FI (14). The variables which made up the FI covered a range of health problems, including comprehensive geriatric assessment (*n* = 7), visual and hearing assessment (*n* = 2), walking and balance function (*n* = 6), diseases and medications (*n* = 16), activities of daily living (ADL and IADL) (*n* = 2), cognition and emotion (*n* = 3), and depression assessment (*n* = 1). At the same time, the variables were coded according to their types following the standard steps (14). For the binary variables, the presence of a deficit was coded as “1” and its absence was coded as “0.” For the multilevel variables, intermediate response level was coded using an additional value to grade variables between “0” and “1,” each lower rating of health was coded to represent a larger deficit (see Appendix 1). The FI is calculated using the formula: FI = number of health deficit scores/total number of items considered as health deficits (i.e., 37). The FI value ranges from 0 to 1. The larger the value, the more health deficits the individual has, i.e., the greater degree of frailty.

### Definition of Follow-Up Outcomes

Based on the standardized questionnaire, death events among the cohort were collected at follow-up in 2020, including whether the subject died, and the time and place of death. For subjects who died during the follow-up period, the staff used standard forms to confirm or obtain information or medical records related to their deaths from family members, local residential committees, or local police stations. A precision method was used for the follow-up duration. If the subject died during the follow-up, the follow-up duration was calculated as (death date–baseline date)/12; if the subject was still alive, it was calculated as (last follow-up date–baseline date)/12.

### Quality Control

Quality controls were employed over the entire course of the baseline survey. The survey design (including the design of study protocol and questionnaire) was certified by experts and revised and validated many rounds using pre-survey. All investigators were medical professionals, and a uniform survey program was used for the training and qualification of investigators prior to the survey. The entire procedure of the survey was supervised and directed by the principal investigator, and each questionnaire was checked and reviewed. The data were double-entered by specially-assigned personnel and strictly checked for logic.

### Statistical Analysis

SPSS 24.0 and Matlab 2020 software were used for data analysis and plotting, and the missing values of variables in the database were imputed using the MCMC method, one of the multiple imputation methods (15). Measurement data were expressed as *x* ± *s*, and the independent sample *t*-test was used for comparison between two groups. Enumeration data were expressed as the number of cases (percentage), and the χ^2^ test was used for comparison between groups. Nonlinear regression techniques were used to fit age-specific frailty index values as a function of age (an exponential function) and to fit the probability of death as a function of the frailty index (a logistic function). The Cox regression model was used to evaluate the hazard ratio (HR) for the impact of FI on mortality in the elderly population of different genders. In the multivariate analysis, as described by Kulminski et al. (16) the FI value was converted into 1% units, i.e., the FI value of each elderly subject was multiplied by 100 and rounded to the nearest whole number. The result obtained indicated the risk caused by each 1% increase in FI values. The Kaplan–Meier method was used to plot the 11-year follow-up survival curves of elderly subjects with different degrees of frailty and then tested using the log-rank method. *P* < 0.05 was considered statistically significant.

## Results

### General Comparison in the Elderly Population of Different Genders

A total of 1,246 elderly subjects were finally included in the analysis, including 519 men (41.7%) and 727 women (58.3%), aged 60–101 years, with an average of 72.05 ± 4.52 years. The educational level of men was higher than women. In terms of health status, women suffered from more chronic diseases, and their IADL score was higher than men. However, there were no significant differences in the types of medication and ADL scores between the elderly of different genders. Compared with women, men had higher MMSE scores and lower CES-D scores, i.e., the cognitive function and depression status of men were better than women. Women had higher FI values than men, i.e., women had a higher degree of frailty than men. There were no significant differences in average age, marital status, or employment status between the elderly of different genders (all *P* > 0.05; see Table 1). As of 2020, a total of 476 subjects died, including 227 men (47.7%) with an 11-year mortality rate of 43.7% (227/519), and 249 females (52.3%) with a 11-year mortality rate of 34.3% (249/727). The mortality rate was higher in men than in women (χ^2^ = 11.546, *P* = 0.001).

**Table 1:** Baseline characteristics of the study sample as separated by sex.

**Variables**	**Men (*n* = 519)**	**Women (*n* = 727)**	***t*/*x*^**2**^**	***P*-value**
Age (*x ± s*)	71.74 ± 4.41	72.16 ± 4.78	1.579	0.115
Years of education [*n* (%)]			152.645	<0.001
0	29 (5.6)	235 (32.3)		
1~6	198 (38.2)	273 (37.6)		
≥7	292 (56.3)	219 (30.1)		
Marital Status [*n* (%)]			0.995	0.319
Married or cohabiting with spouse	279 (53.8)	370 (50.9)		
Others^a^	240 (46.2)	357 (49.1)		
Employment status [*n* (%)]			2.355	0.125
Working	27 (5.2)	25 (3.4)		
Retied	492 (94.8)	702 (96.6)		
*N* of chronic diseases (*x ± s*)	2.16 ± 1.02	2.45 ± 1.18	4.521	<0.001
*N* of medication (*x ± s*)	1.14 ± 1.03	1.17 ± 1.05	0.501	0.616
ADL score (*x ± s*)	95.77 ± 11.07	94.85 ± 10.73	1.472	0.141
IADL score (*x ± s*)	2.49 ± 0.19	3.42 ± 0.17	90.613	0.000
MMSE score (*x ± s*)	25.51 ± 3.49	23.24 ± 4.21	10.061	0.000
CES-D score (*x ± s*)	6.24 ± 4.08	7.16 ± 4.37	3.766	<0.001
FI (*x ± s*)	0.12 ± 0.09	0.14 ± 0.11	3.407	<0.001

*ADL, Activities of daily living; IADL, Instrumental activity of daily living; MMSE, Mini-mental status examination; CES-D, Center for epidemiologic studies depression scale; FI, frailty index*;

a *Including single, separated, divorce, and widowed*.

### Relationship Between Age and the Mean Value of FI in the Elderly Population of Different Genders

**Figure 1:**
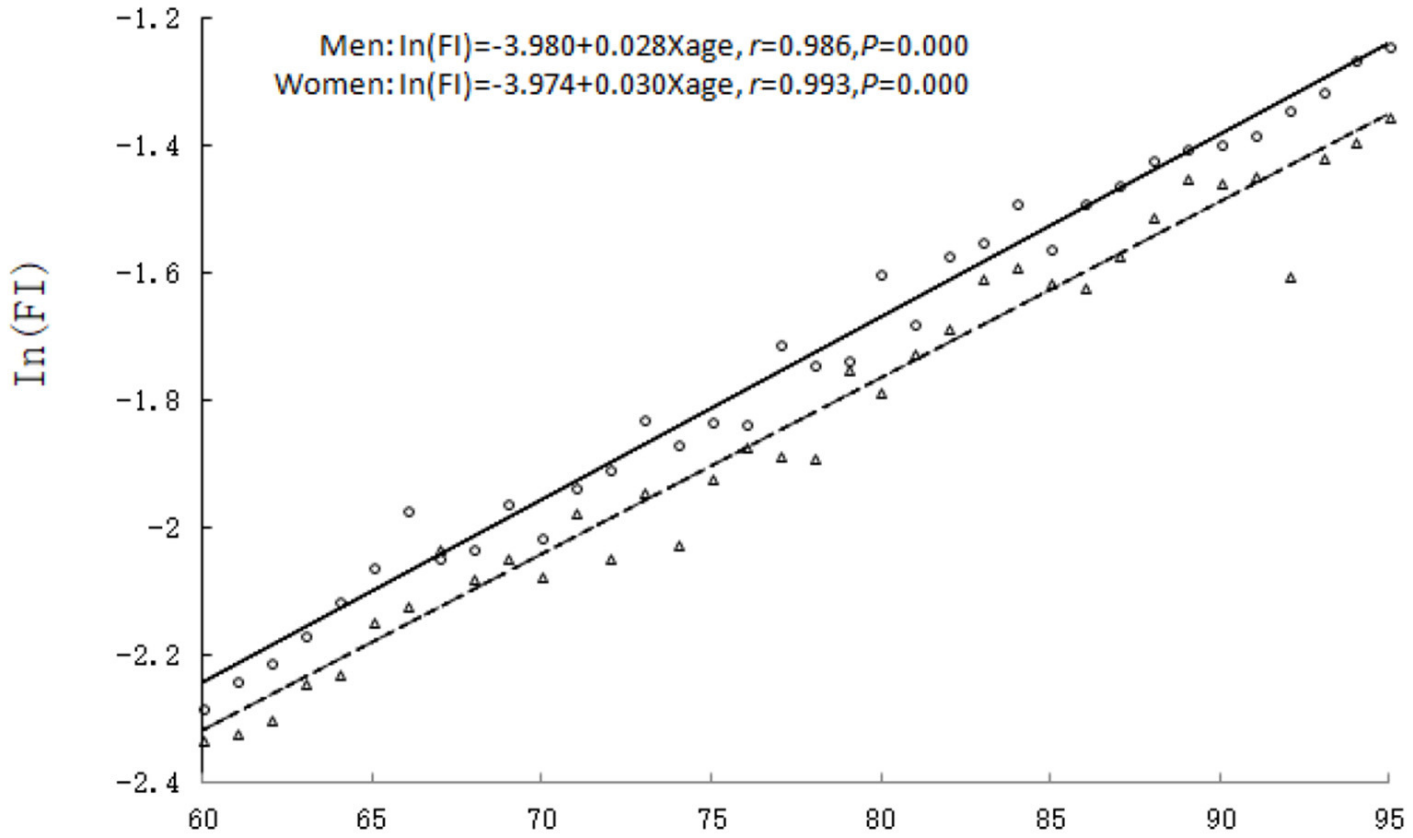
The relationship between age and the mean value of FI. Men: triangle and dashed line; women: circles and solid line.

The relationship between age and the mean value of FI in the elderly population of different genders was analyzed. The results showed that the mean value of FI increased exponentially with age regardless of genders: In(FI) = A + B × Age, where, In(FI) = −3.980 + 0.028 × Age for men and In(FI) = −3.974 + 0.030 × Age for women. The correlation coefficient between age and the logarithm of the FI was high for both men (*r* = 0.986, *P* = 0.000) and women (*r* = 0.993, *P* = 0.000). The FI values of women were higher than men at any age, i.e., women had a higher degree of frailty than men. On a logarithmic scale, the average annual relative increase rate of health deficits and FI values of women was higher than men (*B* = 0.030 vs. 0.028, *t* = 4.137, *P* = 0.023), i.e., the accumulation rate of health deficits in women was faster than men (see Figure 1).

### Comparison of Mortality Rates in the Elderly Population of Different Genders With Different Degrees of Frailty

**Figure 2:**
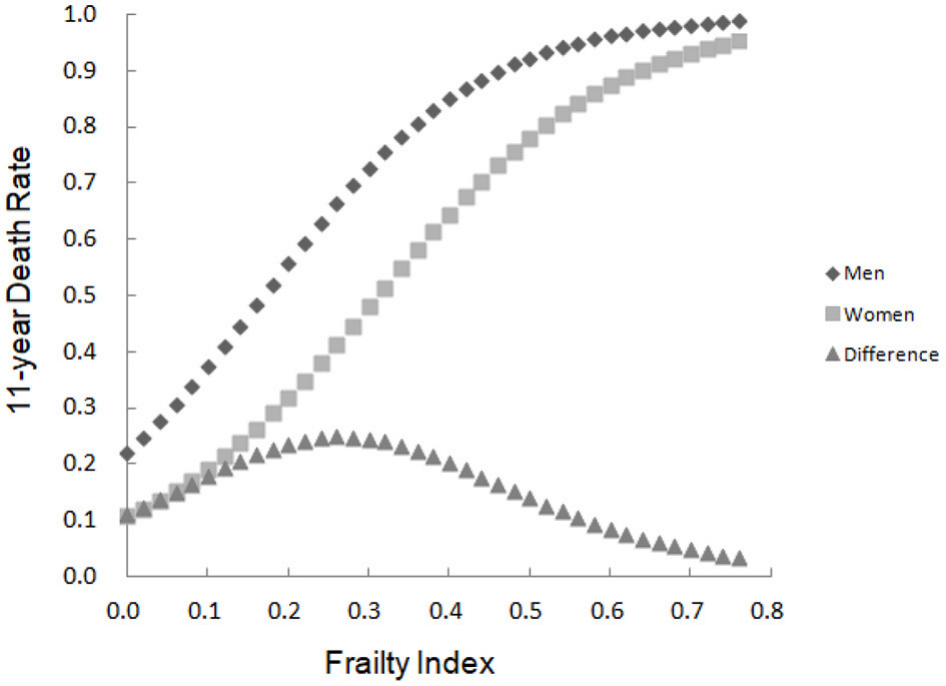
The 10-year death rate as a function of the FI and the mortality difference between men and women.

The trend of mortality rates with FI values in the elderly population of different genders was analyzed. The results showed that the FI was highly related to mortality, which was greater in men than in women. In other words, although women had more deficits than did men, the deficits were less lethal. The difference of the mortality rates between both genders with FI value of 0.2~0.4 was higher than that with other FI values, with the peak occurring with FI value of 0.26, i.e., the difference of mortality rate between both genders was the largest with FI value of 0.26 (see Figure 2).

### Multivariate Cox Regression Analysis of the Impact of FI on Mortality Risk in the Elderly Population of Different Genders

A multivariate Cox proportional hazards model was performed to evaluate the effect of FI on mortality after testing for the proportionality assumption. The results showed that after the adjustment for age, gender (men = 0, women = 1), years of education (0 years = 1, 1–6 years = 2, ≥7 years = 3), marital status (married or cohabiting with spouse = 1, other = 2), employment status (working = 1, retired = 2), the higher the FI value was associated with the higher the mortality risk, irrespective of the overall population or both genders, i.e., frailty increased mortality risk in the elderly population (all *P* = 0.000). Further comparison of the impact of FI on mortality risk by gender showed that the FI value had a greater impact on the mortality risk in men (*HR* = 1.171, 95%*CI*: 1.139–1.249) than women (*HR* = 1.119, 95%*CI*: 1.039–1.137). The FI also had a greater impact on the mortality risk than age (*HR* = 1.145 vs. 1.048; see Table 2).

**Table 2:** Multivariate Cox regression analysis of the impact of FI on 10-year mortality risk in the elderly population of different genders.

**Variables**	**Overall (** ***n*** **= 1,246)**			**Men (** ***n*** **= 519)**			**Women (** ***n*** **= 727)**		
	***B*-value**	***HR* (95%*CI*)**	***P*-value**	***B*-value**	***HR* (95%*CI*)**	***P*-value**	***B*-value**	***HR* (95%*CI*)**	***P*-value**
Age	0.047	1.048 (1.045~1.061)	0.000	0.046	1.046 (1.039~1.058)	0.000	0.049	1.051 (1.047~1.063)	0.000
Sex	−0.439	0.645 (0.581~0.692)	0.000	—	—	—	—	—	—
Year of education	−0.081	0.922 (0.854~0.987)	0.032	−0.047	0.954 (0.871~0.965)	0.027	−0.119	0.888 (0.739~1.013)	0.054
Working status	−0.138	0.871 (0.629~1.347)	0.584	−0.172	0.842 (0.528~1.722)	0.342	−0.124	0.883 (0.817~1.211)	0.865
Marriage status	0.276	1.318 (1.179~1.452)	0.046	0.416	1.516 (1.324~1.951)	0.037	0.112	1.119 (0.817~1.193)	0.173
FI	0.135	1.145 (1.065~1.217)	0.000	0.158	1.171 (1.139~1.249)	0.000	0.112	1.119 (1.039~1.137)	0.000

*FI, frailty index*.

### Comparison of Kaplan–Meier Curves in the Elderly Population of Different Genders With Different Degrees of Frailty

**Figure 3:**
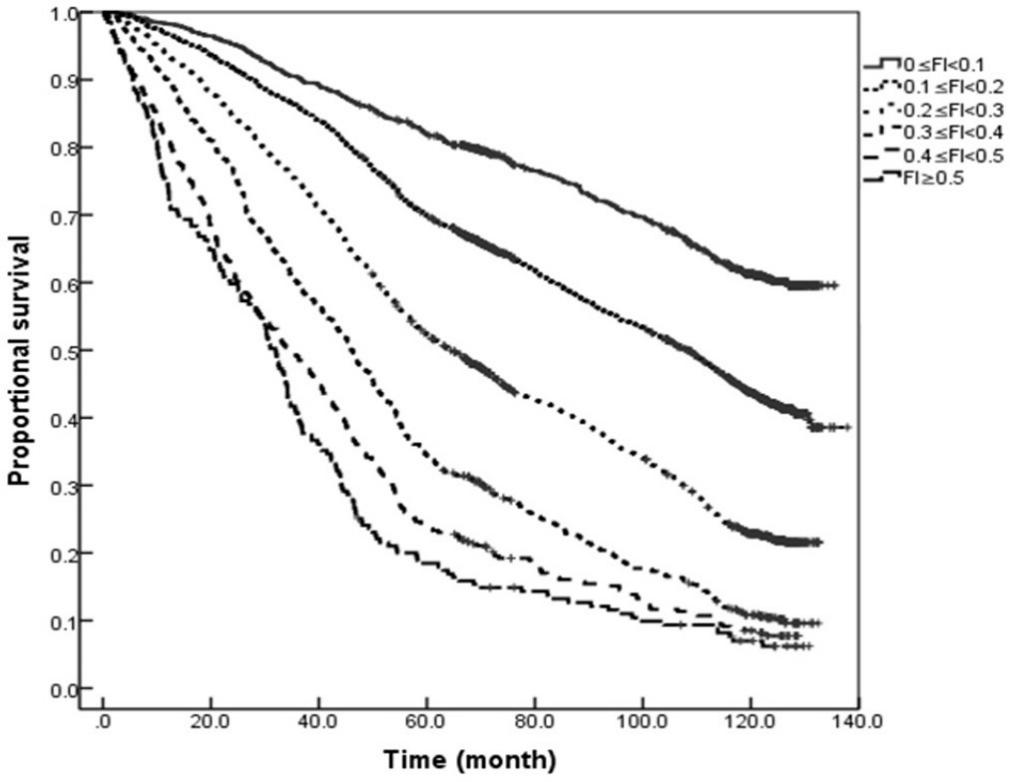
Kaplan–Meier curves for the proportional survival of older men with different degrees of frailty.

**Figure 4:**
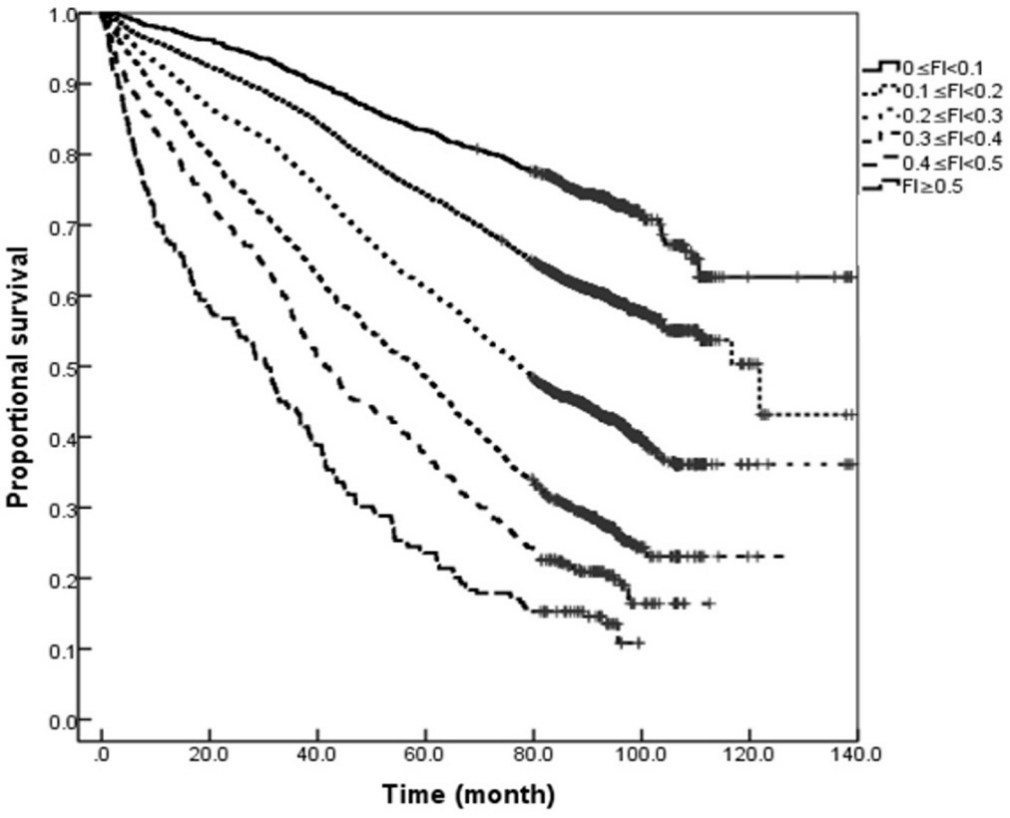
Kaplan–Meier curves for the proportional survival of older women with different degrees of frailty.

The results of Kaplan–Meier analysis showed that the median survival for women followed up for 11 years was 95.26 months, which was higher than the 89.52 months for men (*Log-rank* = 9.249, *P* = 0.002). Further comparison of the Kaplan–Meier curves for the elderly population of different genders with different degrees of frailty (0 ≤ FI < 0.1, 0.1 ≤ FI < 0.2, 0.2 ≤ FI < 0.3, 0.3 ≤ FI < 0.4, 0.4 ≤ FI < 0.5, and FI ≥ 0.5) showed that increasing grades of the FI showed a dose-response effect in relation to survival for both men and women. The median survival with FI values of 0.1 ≤ FI < 0.2, 0.2 ≤ FI < 0.3, 0.3 ≤ FI < 0.4, 0.4 ≤ FI < 0.5, and FI ≥ 0.5 were 108.11, 64.34, 46.00, 35.30, and 31.95 months for men, and 121.82, 77.53, 58.25, 41.38, and 30.58 months for women, respectively, i.e., the survival of women was longer than men with FI values of 0.1 ≤ FI < 0.2, 0.2 ≤ FI < 0.3, 0.3 ≤ FI < 0.4, and 0.4 ≤ FI < 0.5, and the difference was statistically significant (all *P* < 0.05). However, the difference in the survival rates between both genders with severe frailty (FI ≥ 0.5) was not statistically significant (*P* > 0.05; see Figures 3, 4).

## Discussion

In recent years, more and more geriatric researchers have begun to investigate the frailty in the elderly population. The systematic review has demonstrated that the FI is the only assessment tool that covers all factors related to frailty. It is the most useful tool for assessing frailty in the elderly population in routine care and community settings (9). In 2009, with the support from the China–Canada Collaboration on Aging and Longevity (CCCAL) (17), our team comprehensively and systematically utilized the FI to evaluate the health status of the elderly population in Mainland China and verify the applicability of the FI assessment tool in Chinese elderly population (18–20). In this study, our team used the FI model to assess frailty in the elderly population based on a follow-up survey of Beijing urban communities and analyzed the differences in the degree of frailty between elderly men and women, as well as the impact of frailty on mortality risk. The findings showed that the FI value increased exponentially with age for both genders and elderly women exhibited a higher degree of frailty than men at any age, i.e., elderly women had a faster accumulation rate of health deficits than men. It has been reported that the FI increases exponentially rather than linearly with age, and on a logarithmic scale, the average annual relative increase rate is about 3% (18), which is similar to the values of 3.0% for elderly men (*B* = 0.030) and 2.8% for elderly women (*B* = 0.028) in this study. It is noteworthy that the results of this study also showed that women had a higher degree of frailty than men, however, the mortality rate of men is higher than women for any degree of frailty, with the greatest difference at the mild to moderate degrees of frailty (FI = 0.26). That is, the cumulative risk of health deficits for elderly men is higher than for elderly women, especially for those with mild to moderate degrees of frailty. This conclusion has been verified in different countries, as well as in urban and rural areas of the same regions (21–24). This result also confirms the consensus that “men live better and women live longer.” There is no definite conclusion on the specific mechanism. Some researchers believe that changes in sex hormone levels during aging are involved in the occurrence of frailty in the elderly population. The decline of sex hormone levels is directly related to muscle weakness, fatigue, and impaired function in the elderly population. One study suggested that frailty in elderly men was closely related to androgen levels, and high levels of free testosterone and dihydrotestosterone in serum reduced the risk of frailty in elderly men (25). In general, elderly women are more likely to exhibit various symptoms of frailty than elderly men. However, from a psychological perspective, men have a higher threshold for tolerating dysfunction and exhibit better physiological function, but the health reserves of elderly men are poorer than women. Therefore, the accumulated health deficits are more fatal to elderly men (26).

In order to further clarify the impact of FI on mortality risk in this study, a Cox regression analysis was performed after the adjustment for confounding factors, such as age, gender, and years of education. The results showed that greater FI value was associated with greater mortality risk for both genders. That is, frailty increases the mortality risk in the overall elderly population, and the FI value has a greater impact on mortality risk in elderly men than elderly women. Through the relative heterogeneity analysis of the degree of frailty in men and women, Yang et al. (26) pointed out that the ability of women to respond to external stimuli was more decreased with age than men, but at the same time, women were better able to resist adverse stimuli than men. Therefore, compared with men of the same age, women of frailty could more effectively retain their own responsiveness and maintain the system integrity for combating adverse environmental stimuli. This difference may also partially explain the higher health expectancy in women. At present, more and more scholars have begun to investigate the biological age of individuals, and findings show that the biological age of individuals is more effective in predicting adverse health outcomes, such as death (27–29), than the chronological age. This study also demonstrates that the FI value is a better indicator to predict death than age. The FI is also more closely related to the biological age of individuals than the chronological age, and the correlation has been used to calculate and determine the biological age of individuals by fitting the functional relationship between the age and the FI. Thus, compared with the chronological age, the FI can better reflect the age-related changes in the health status of individuals (30). In this study, the survival analysis of the elderly population with different degrees of frailty revealed that in severe frailty (FI ≥ 0.5), the superior survival rate of elderly women over elderly men disappeared. It is consistent with the study results reported by Yang et al. (26) that the difference change between genders decreased with the increase of age and degree of frailty. Therefore, the degree of frailty should be considered when conducting interventions for frailty in the elderly of different genders.

The following limitations of this study should be noted. Firstly, the baseline data were obtained based on the questionnaire, and there may be reporting bias, or recall bias because elderly subjects due to differ in understanding or recalling the content in the questionnaire. Therefore, in the course of training, it is essential for investigators to conduct the survey patiently and meticulously with both the individual subjects and their family members or nursing staff, so as to obtain detailed and true information as far as possible. Secondly, during the prospective observation period, the lost to follow-up subjects who left or moved from the survey site were all comparatively younger, and the lost to follow-up bias caused a certain impact on the results. Thirdly, this study did not collect causes of death, and the impact of other factors besides frailty on death cannot be ruled out. Moreover, although some objective physical function directors (static balance test, dynamic balance test, 5 times sit-to-stand test, up-and-go test) were included in the questionnaire, other important indicators that are strongly related to frailty, such as handgrip strength and walk speed, were not covered in the questionnaire due to practical limitation during the survey. The questionnaire requires future improvement and supplement with relevant information for more in-depth analysis.

## Conclusion

In this study, the FI model was used to assess the frailty of the elderly population in the community of Beijing. The results are consistent with those studies conducted in developed countries. The FI can better reflect the characteristics of frailty and is suitable to assess the health status or predict adverse outcomes in the elderly population. The study has once again verified the effectiveness of the FI model in the elderly population in China. The frailty of elderly men and elderly women, and the impact of frailty on mortality are various. Therefore, specific interventions for frailty should be conducted in the elderly population of different genders, as well as of different degrees of frailty.

## Data Availability Statement

The original contributions presented in the study are included in the article/supplementary material, further inquiries can be directed to the corresponding author/s.

## Ethics Statement

Ethical review and approval was not required for the study on human participants in accordance with the local legislation and institutional requirements. The patients/participants provided their written informed consent to participate in this study.

## Author Contributions

JS conducted the survey, performed statistical analysis of the data, and drafted the paper. YT helped with the analysis and assisted with manuscript preparation. LM and BZ conducted the survey and collected the data. CD assisted data analysis and result interpretation. HX and PY initiated and designed the study, revised the paper, and finally approved the version to be published. All authors read and approved the final version of the manuscript.

## Conflict of Interest

The authors declare that the research was conducted in the absence of any commercial or financial relationships that could be construed as a potential conflict of interest.

## Publisher's Note

All claims expressed in this article are solely those of the authors and do not necessarily represent those of their affiliated organizations, or those of the publisher, the editors and the reviewers. Any product that may be evaluated in this article, or claim that may be made by its manufacturer, is not guaranteed or endorsed by the publisher.

## Appendix

**Table A1:** Variables for calculating the frailty index and their codes.

**Variables**	**Codes**
**Comprehensive geriatric assessment**	
Falls	No = 0, Yes = 1
Urinary incontinence	Never = 0, 1 time/week or less = 0.25, 2-3 times/week = 0.5, About 1 time/day = 0.75, Several times/day = 1
Pain	None = 0, Tolerable without affecting any activities = 0.33, Tolerable but affecting some activities = 0.66, intolerable but still able to make phone calls/watch TV/perform other activities = 1
Constipation	No = 0, Yes = 1
Weight loss	No = 0, Yes = 1
Sleep disorder	No = 0, Yes = 1
Usage of sleep aids	No = 0, Yes = 1
**Visual and hearing assessment**	
Vision	>4 m = 0, 1–3 m = 0.5, 1 m, or less = 1
Hearing	Completely clear = 0, Not very clear = 0.5, Not clear at all = 1
**Walking balance function**	
Usage of walking aids	No = 0, Yes = 1
Walking 400 m independently	Yes = 0, No = 1
Static balance test	> 10 s = 0, ≤ 10 s = 0.5, Unable to complete = 1
Dynamic balance test	> 10 s = 0, ≤ 10 s = 0.5, Unable to complete = 1
5 times sit-to-stand test	<10 s = 0, ≥10 s = 0.5, Unable to complete = 1
Up-and-go test	<12 s = 0, ≥12 s = 0.5, Unable to complete = 1
**Diseases and medications**		
Chronic diseases(15 types)^a^	Each no = 0, Each yes = 1
Number of medications	Infrequent medications = 0, 1–3 medications = 0.5, ≥4 = 1
**Assessment of activities of daily living**	
ADL	100 points = 0, 75–95 points = 0.25, 50–70 points = 0.5, 25–45 points = 0.75, 0–20 points = 1
IADL	≤ 5 points = 0, >5 points = 1
**Cognition and emotion**	
Memory is diminished	No = 0, Yes = 1
Emotional instability	Never = 0, Sometimes = 0.5, Often = 1
MMSE^b^	Cut-off value is normal = 0, Below cut-off is a cognitive deficit = 1
**Depression assessment**	
CES-D	<10 points = 0, ≥10 points = 1

*ADL, activities of daily living; IADL, instrumental activities of daily living; MMSE, mini-mental state examination; CES-D, Center for Epidemiological Studies-Depression Scale (simplified version)*;

a *The 15 types of chronic diseases include hypertension, heart disease, diabetes, anemia, hyperlipidaemia, sleep apnea syndrome, gastrointestinal disease, cerebrovascular disease, dementia, tumor, protrusion of intervertebral disc, thyroid disease, osteoporosis, osteoarthritis, arthrolithiasis*;

b *MMSE cut-off value is related to the level of education, 17 points for no education, 20 points for <6 years of education, 24 points for more than 6 years*.
